# The distal metatarsal screw is not always necessary in third-generation MICA: a case–control study

**DOI:** 10.1007/s00402-022-04740-7

**Published:** 2022-12-29

**Authors:** Norbert Harrasser, F. Hinterwimmer, S. F. Baumbach, K. Pfahl, C. Glowalla, M. Walther, H. Hörterer

**Affiliations:** 1 Clinic of Orthopaedics, Klinikum rechts der Isar, Technical University Munich, Ismaningerstr. 22, 81675 Munich, Germany; 2 Department of Orthopaedics and Trauma Surgery, Musculoskeletal University Center Munich (MUM), University Hospital, LMU Munich, Marchioninistraße 15, 81377 Munich, Germany; 3 FIFA Medical Centre, Center for Foot and Ankle Surgery, Schön Clinic Munich Harlaching, Harlachinger Straße 51, 81547 Munich, Germany

**Keywords:** Chevron osteotomy, Hallux valgus, Beveled screw, Forgotten bunion

## Abstract

**Introduction:**

To evaluate the clinical and radiological results after fixation of the first metatarsal head (MTH) with one or two screws as part of the third-generation minimally invasive Chevron–Akin osteotomy (MICA) for hallux valgus deformities.

**Materials and methods:**

Between August 2020 and November 2021, 55 MICA procedures (50 patients, male:female = 7:43), 22 with two (MICA2), 33 with one screw (MICA1) were performed for mild to severe hallux valgus deformities. Exclusion criteria were a concomitant pes adductus (Sgarlato angle > 20°) or hindfoot/midfoot deformities requiring treatment. In 27 cases, additional procedures on the forefoot (small toe corrections or metatarsal osteotomies II–V) were necessary. Pre- and post-operatively, hallux valgus angle (HVA) and intermetatarsal I/II angle (IMA) were measured. Clinically, subjective satisfaction, range of motion (ROM) of the first metatarsophalangeal joint (MTPJ), and pain level (NRS score) were evaluated. The minimum follow-up was 12 months.

**Results:**

Displacement of MTH was 70–90% on average, all osteotomies showed full consolidation at latest follow-up. In one case of either group, a slight subsidence of MTH was documented. The radiological and clinical parameters showed no differences between the groups. The pain level improved by an average of three points. The mobility of the MTPJ showed a slight reduction in nine cases after three months (4 MICA2, 5 MICA1) which persisted in three cases. Fifty-two of 55 patients (95%) would opt again for the operation.

**Conclusions:**

Fixation of the first MTH with a single bicortical screw in MICA with moderate lateralization of MTH shows stable anchoring and good clinical results. The routine use of a second metatarsal screw can be omitted.

## Introduction

Hallux valgus surgery can achieve good to very good results in over 90% of cases with modern, open procedures [7]. Classically, distal and diaphyseal osteotomies are used for mild to moderate deformities; the remaining deformities can be corrected with proximal osteotomies or a modified Lapidus arthrodesis. The minimally invasive Chevron/Akin osteotomy (MICA) is a modern, third-generation MIS (minimally invasive surgery) procedure [2, 3, 15, 16]. Due to the possibility of extreme displacement of the first metatarsal head (MTH), the indication for MICA can be expanded to correct even severe hallux valgus deformities [18]. Technically, the percutaneous osteotomy is performed at a subcapital and extra-articular level of the first metatarsal, whereby the osteotomy shape can be straight or Chevron-like with a plantar leg. The first MTH is usually fixed with two screws in the desired position (lateralization of more than 100% of the shaft width is possible), whereby in the classic technique according to Redfern/Vernois the proximal screw runs bicortically, the distal one mostly monocortically [12].

**Fig. 1:**
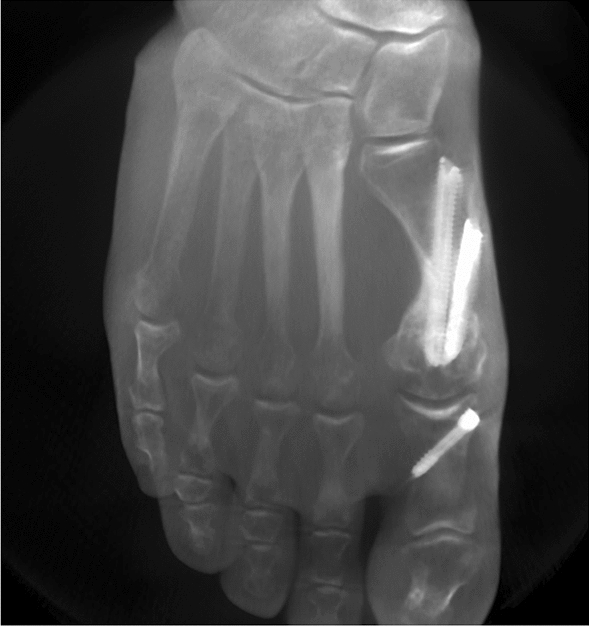
Consolidated MICA with 2 screws. The prominent distal metatarsal screw head was prominent and disturbing the patient. The conflict was resolved by metal removal and resection of the residual bump

Although excellent results can be achieved with MICA, there are also typical complications such as prolonged postoperative swelling of the foot, painful protracted secretions of the portals due to residual bone debris and sometimes disruptive foreign material [9]. The distal screw, which is inserted to secure against rotation, is particularly at risk here, since the soft tissues in this area of the metatarsal shaft are thin. Additionally, the screw head can sometimes limit the resection of the metatarsal protrusion or even be exposed after that (Fig. 1). Despite the adjusting screws with beveled screw heads that are now frequently used, a disruptive material conflict can impair the result.

Clinical experience suggests, there is usually sufficient stability in the area of the metatarsal osteotomy if one bicortical screw is inserted and translation of the MTH is less than 100% of the shaft width [5]. Hence, the routine insertion of the second monocortical screw, as described in the classical technique, could be omitted. To the best of our knowledge, no data dealing with this problem have been published so far. Therefore, the aim of the present work was to analyze the clinical–radiological results after MICA with and without the use of a second screw. The working hypothesis was that there would be no significant differences between the groups regarding radiographic outcome but less additional procedures for screw removals would be necessary if the second screw has been omitted.

## Patients and methods

The present study was designed as a retrospective analysis with prospective follow-up of our institutional foot and ankle registry data. The study was conducted as a proof-of-concept in accordance with the declaration of Helsinki and was approved by the local ethical review board (Technische Universität München, 2022–503-S-KH). All patients with symptomatic mild to severe hallux valgus deformity (Hallux valgus angle [HVA] > 20°, intermetatarsal angle I/II [IMA] > 10°) and correction with MICA in the period between August 2020 and November 2021 were included (Table 1). The MIS Chevron was fixed with either one (MICA1) or two (MICA2) screws, depending on the surgeon’s perception of intraoperative stability at the osteotomy site.

**Table 1:** Patient characteristics of the two groups; Chevron osteotomy as part of the MICA fixed with one (MICA1) or two (MICA2) screws

	MICA1	MICA2	*p* value
Patients/feet	30/33	20/22	0.051
Age, mean	52,4	45	0.067
Gender, female/male	27/3	16/4	0.039
Side, left/right	10/23	10/12	0.044
Additional procedures (feet)	15	12	0.68

All operations were performed by two fellowship trained foot and ankle surgeons with more than five-year experience with MIS techniques (NH, HH). Patients with a follow-up of < 12 months after surgery, a concomitant pes adductus (Sgarlato angle > 20°) and additional interventions on the midfoot and/or hindfoot were excluded. Pre- and post-operatively, at the different follow-up appointments (3, 6 and ≥ 12 months postoperatively), various clinical and radiographic data were collected. Clinically, the mobility of the first metatarsophalangeal joint (MTPJ) was documented and its difference (pre- to postoperatively) was calculated (< 10°: no significant limitation, 10°–30°: moderate limitation; > 30°: severe limitation). Additionally, the pain level (numeric rating scale NRS, 0 (no pain)–10 (worst possible pain)) was recorded and the patient was asked: “Are you satisfied with the result of the operation?” (Answer options: yes/unsure/no). Satisfied patients were specifically asked: “Are there any restrictions or complaints in the area of the operated hallux at all?” If this question was answered in the affirmative (there are no restrictions/complaints at all), then this was classified as “forgotten bunion” (in accordance to a perfect result after joint arthroplasty [14]). Radiographically, images were taken in a standardized fashion (foot dorso-plantar and lateral under full weight bearing) and the following measures were taken: degree of displacement of the first MTH in percent to the shaft diameter (< 70%, 70–90%, > 90%), HVA and IMA. Additionally, in a qualitative manner, a possible loss of correction and the osseous consolidation in the area of the metatarsal osteotomy were documented.

### Surgical technique

**Fig. 2:**
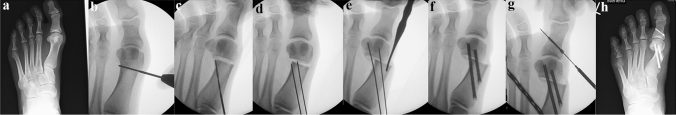
Main surgical steps of the MICA with fixation of the metatarsal osteotomy with two screws. **a** Preoperative X-ray; **b** Osteotomy of the first metatarsal bone at the metadiaphyseal junction with a 2 × 19.5 mm burr; **c** Placement of the proximal, bicortical screw hole with a 2 mm drill (facilitates exact positioning in the area of the distal cortex); **d** placement of the second K-wire for the distal, monocortical screw; **e** Lateralization of the MTH with the raspatory and temporary fixation with the K-wires. With a mild deformity, a displacement of about 75% of the shaft width was considered sufficient; **f** Replacement of the K-wires with corresponding 4 mm fully threaded screws; **g** Akin osteotomy and fixation with 3 mm screw. This is followed by a resection of the pseudoexostosis and the overhang on the medial side of the 1st metatarsal bone; **h** Postoperative X-ray after 6 weeks (additionally, accompanying MIS interventions were performed on the forefoot)

The operation was performed according to previously described techniques [12] under regional or general anesthesia with the use of a thigh torniquet. The first step was the percutaneous extra-articular Chevron osteotomy with a Shannon burr (2 × 19.5 mm). The osteotomy was always aimed for in a V-shape to preserve a plantar limb. A K-wire was then inserted bicortically into the first metatarsal from proximal medial to distal lateral. The proximal metatarsal shaft was entered with a raspatory and the MTH was shifted laterally according to the deformity. The K-wire was then forwarded into the MTH and replaced with a fully threaded screw. The stability of this construct was thereafter critically checked, only if micro-motion was palpable, a second screw slightly distally to the first was inserted. The next step was a percutaneous Akin osteotomy, which was fixed with a 3 mm screw. The metatarsal protrusion was removed in an inside-out fashion through the first portal. If necessary, a percutaneous lateral release [8] and resection of the pseudoexostosis on the MTH were performed (Fig. 2).

### Postoperative care

The same care was recommended for both groups. To avoid excessive swelling, the foot should be elevated frequently for the first 2 weeks. A flat postoperative shoe with a rigid sole (Fa. Darco, DualRelief) was used for 6 weeks, crutches were recommended for two weeks. Redression of the big toe with bandages was performed for 2 weeks, thereafter elastic taping was recommended for further 4 weeks. Full weight bearing of the heel was allowed immediately after the operation, the big toe could be beared after 3 weeks. Sports with high forefoot stress (e.g. jogging) were not allowed for the first 10 weeks.

### Statistical analysis

Normal distribution was verified using D’Agostino–Pearson testing. An independent *t*-test and a Mann–Whitney *U*-test were performed for normal and non-normal distributed data, respectively, to describe significant differences between the groups. Statistical significance was assumed for all *p* values < 0.05. Correlation between different variables was determined using Pearson’s correlation coefficient (PCC). Correlation was classified as minimal (PCC ≤ 0.25), low (0.26 < PCC < 0.5), moderate (0.5 ≤ PCC < 0.7), high (0.7 ≤ PCC < 0.9), and excellent (PCC ≥ 0.9) [11]. All analyses were performed with Python 3.9.6 (https://www.python.org/) and the scipy-library (https://scipy.org/).

## Results

### Clinical results

**Fig. 3:**
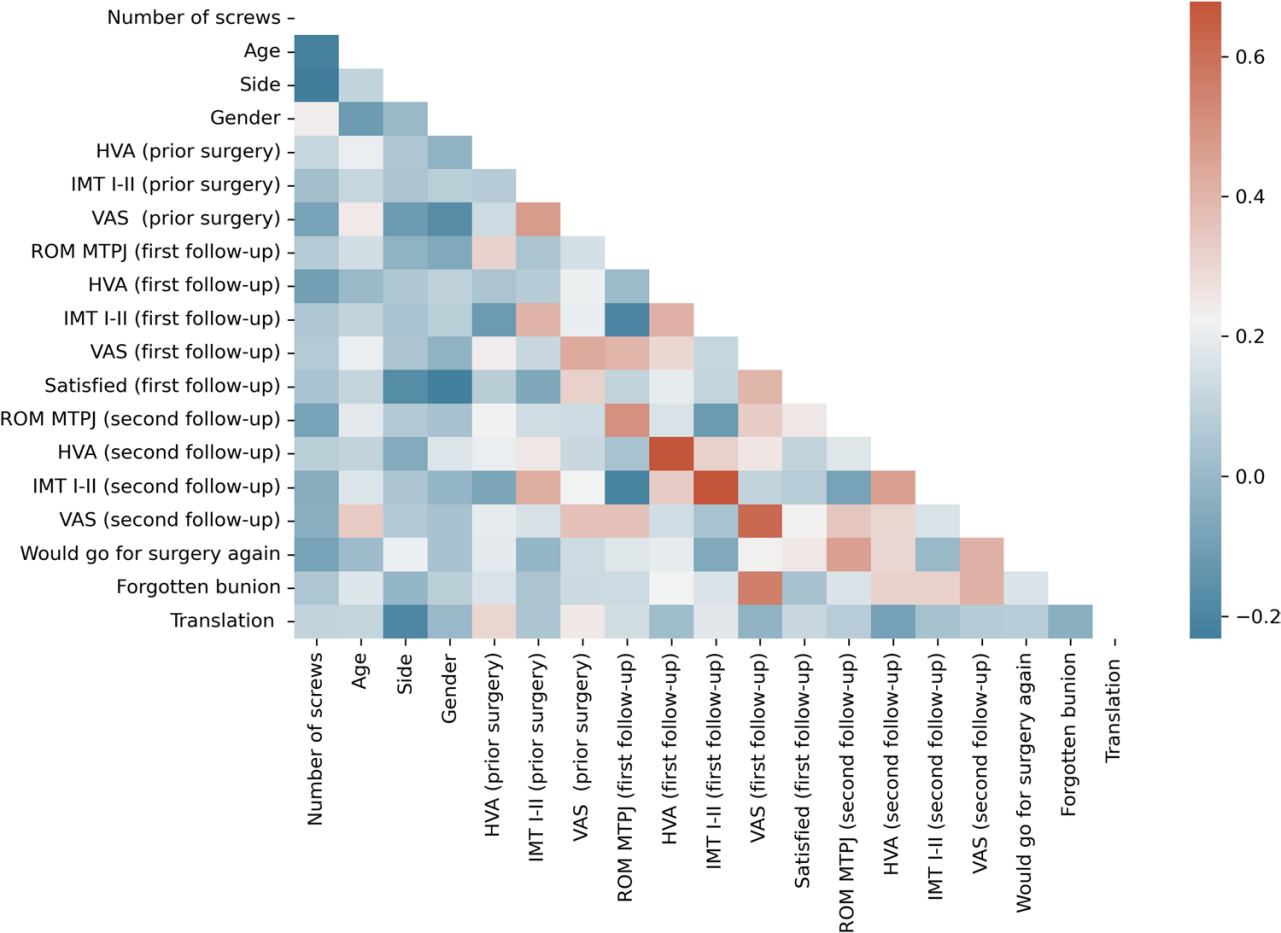
Correlation matrix for MICA 1

**Fig. 4:**
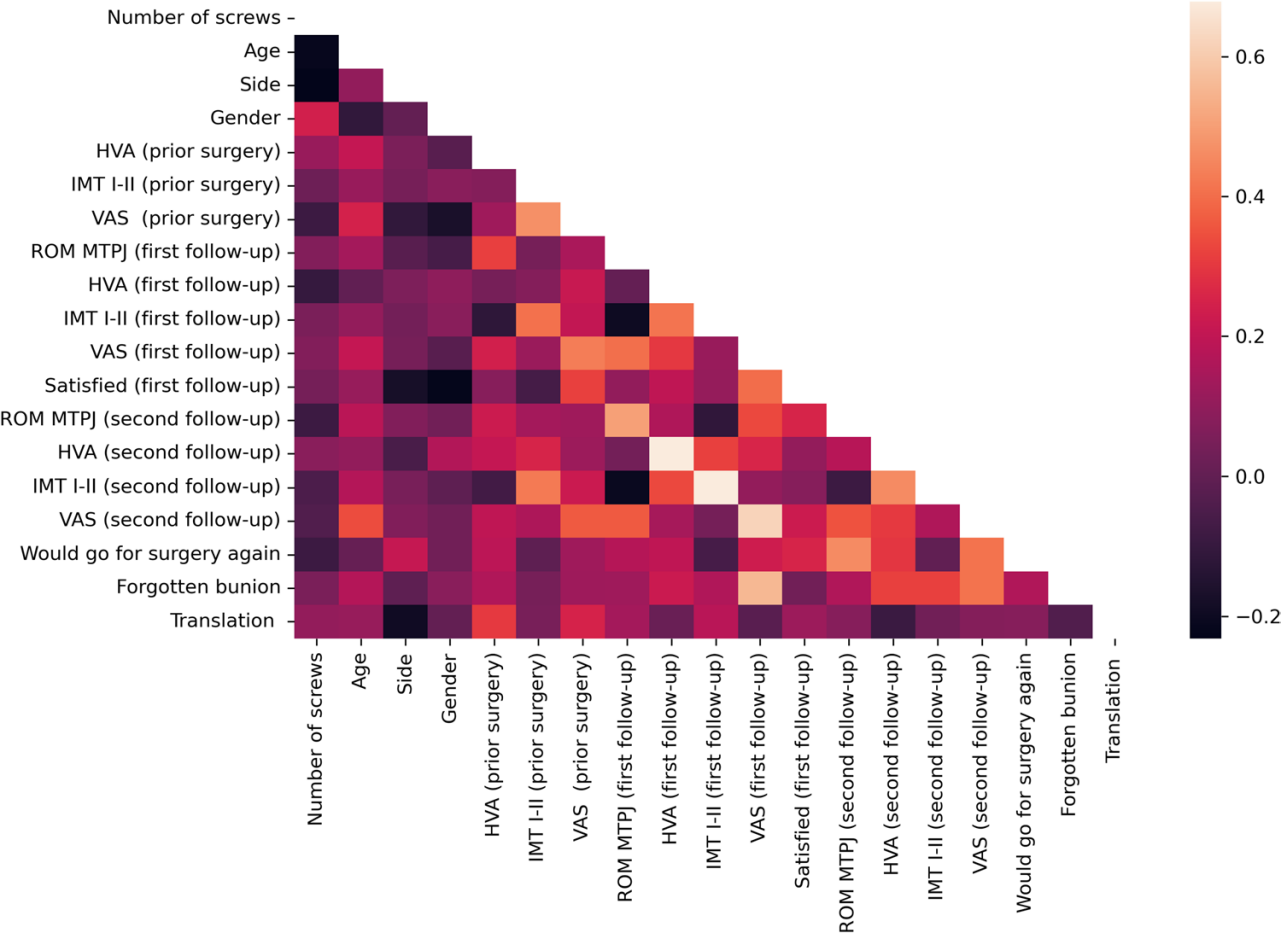
Correlation matrix for MICA 2

Moderate stiffness of more than > 10° in comparison to the preoperative range of motion occurred in 8 (MICA1, 24%) and 4 (MICA2, 18%) cases after 3 months and persisted in two cases in the MICA1 group, and one case (5%) of the MICA2 group (6%) after 12 months. There were no cases of severe stiffness in any of the groups (defined as reduction in ROM > 30 degrees). MICA1 and MICA2 surgeries achieved similar improvements in pain score (mean preoperatively: 3; mean postoperatively after 12 months: 0.5). The number of patients that would agree to undergo the operation again and the degree of satisfaction with the surgery (MICA1: 21/22, 95%; MICA2: 31/33, 94%) were similar in both groups. The rate of forgotten bunion was slightly higher in the MICA1 group (9/33, 27%; 4/22, 18% in MICA2 group). Correlation analysis revealed no high or excellent correlations of any parameters in either group (Figs. 3 and 4).

### Radiologic results

**Fig. 5:**
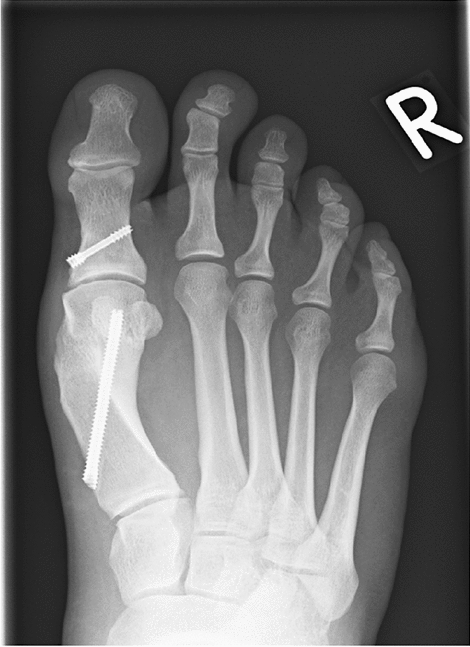
Six month after single-screw MICA with complete healing of metatarsal osteotomy

Translation of the MTH was 70–90% of the shaft width in 29 cases of MICA1 (4 cases < 70%), and 19 cases of MICA2 (2 cases < 70%, 1 case > 90%). All osteotomies healed within the study period (Fig. 5). The HVA decreased from 30.9 to 10.2 degrees in MICA1 and from 30.8 to 11.0 degrees in MICA2. The IMA decreased from 15 to 5.5 degrees in MICA1 and from 14.6 to 5.8 degrees in MICA2.

### Complications

Delayed wound healing occurred in three cases of either group (MICA1:9%; MICA2:14%), healing occurred in all cases with conservative measures. Deep infection was found in one case of MICA2 (5%) at the level of the distal metatarsal screw. Here, removal of the screw, local debridement and systemic antibiotics for 2 weeks were successful. Slight subsidence of the metatarsal osteotomy occurred in 1 case of each group (MICA1: 3%; MICA2: 5%), the final result was regular and both patients were satisfied after 12 months. Recurrence of the deformity with HVA > 20° and/or IMA > 10° occurred in one case of each group (MICA1: 3%; MICA2: 5%). No cases of hallux varus were observed. Significantly more patients were dissatisfied with the implants in MICA2 (7/22, 32%) than in MICA1 (1/33, 3%), in all cases removal of the screw and resection of the bump was successful.

## Discussion

The present study showed no significant differences regarding the clinical–radiological results and consolidation rates in third-generation MICA fixed with either one or two metatarsal screws. Nevertheless, we found a clear tendency to less disruptive material conflict in the single-screw MICA. Taken together, it was clearly shown that MICA is an effective and successful method in correcting hallux valgus deformities in the short-term follow-up, regardless of whether one or two metatarsal screws are used.

Two meta-analyses including numerous publications have been able to demonstrate the effectiveness of MICA in the context of hallux valgus correction [9, 10]. Compared to open procedures, MICA shows concordantly lower postoperative pain levels and improved mobility of the first MTPJ, especially in the first three postoperative months. Nevertheless, typical complications associated with MICA have been described. This includes prolonged postoperative swelling and bothersome screw heads medial on the first metatarsal shaft. Especially disruptive osteosynthesis material after MICA was described in up to 24%, whereas it is only reported in up to 2% after open scarf osteotomy [6]. Our findings are in accordance with these data, since 32% of patients in the MICA2 group underwent screw removal at least of the distal screw. Material discomfort mainly affects the distal metatarsal screw, since the soft tissue is particularly narrow in this area. Even though this complication can be solved without major efforts, it should clearly be prevented. In this respect, some technical notes must be considered: Taking X-rays exactly tangentially to the medial plane of the first metatarsal after screw insertion gives important information regarding insufficient insertion of the screws; additionally, the use of screws with a beveled screw head can further diminish the risk. Another problem, rather specific for the distal screw, is that it sometimes limits the resection of the metatarsal protrusion that occurs after lateral displacement of the MTH, especially if the proximal screw is inserted distally. A disruptive pseudoexostosis can persist at the level of the metatarsal osteotomy that has to be resected (in conjunction with screw removal) in a second step after consolidation of the osteotomy (Fig. 1). The results of the present study show that if the osteotomy is classified stable intraoperatively after insertion of one bicortical screw, the insertion of the distal metatarsal screw can be dispensed without jeopardizing the consolidation. This finding is not new, since several authors reported already on MICA techniques, where positioning of a single metatarsal screw was sufficient to stabilize the osteotomy [4–6]. Nevertheless, in the quoted papers, only images with single-screw MICA (or modified techniques of MIS hallux corrections) are pictured and no scientific work-up of this technique was conducted. Hence, to the best of our knowledge, the present study is the first to prove that a single metatarsal screw is often enough to stabilize the Chevron osteotomy in third-generation MICA. Although in two cases a slight subsidence of the MTH could be observed, this phenomenon has also been observed when using two screws and is partly due to reduced bone quality or forced loading in the early postoperative period. Nevertheless, in the present study, a 100% consolidation rate could be determined in the MICA1 group, and the final result was not negatively affected by the subsidence of the MTH. Experience suggests the amount of stability at the metatarsal osteotomy site can be augmented, and thus the risk of subsidence be diminished, if a Chevron-type cut is used. This is in contradiction to findings of a cadaver study where in a cantilever bending model, no significant differences in load to failure, yield load, and stiffness between transverse and Chevron-type osteotomy techniques were found [1]. Nevertheless, in this quoted study, the metatarsal osteotomies were fixed with two metatarsal screws in either group; therefore, no conclusion could be drawn, whether one screw in the Chevron-type group would be as stable as two screws. Additionally, the load to the MTH was applied perpendicularly to the shaft axis, which is unphysiological with respect to the first metatarsal pitch. Taken together, the authors of the present study believe a Chevron-type cut is more suitable if only one screw is used, and therefore aim for this type of cut whenever possible. Nevertheless, further studies are needed dealing with this topic.

The primary goal of hallux valgus corrective surgery is to align the first toe and thus regain physiologic loading within the MTPJ. HVA was corrected from 30.9 to 10.2 degrees in MICA1 and from 30.8 to 11.0 degrees in MICA2. IMA decreased from 15 to 5.5 degrees in MICA1 and from 14.6 to 5.8 degrees in MICA2. These corrections are reported to the same amount from other authors performing third-generation MICA [10, 13]. Hence it can be stated that the goal of realignment was achieved in nearly all patients, regardless the number of metatarsal screws: both groups reported the same decrease in pain after operation and nearly all patients would opt to undergo the surgery again.

As already mentioned, one important advantage of MICA in comparison to open techniques are lower rates of soft tissue complications and less stiffness of the first MTPJ [13] Stiffness after MICA is reported anecdotally after third-generation MICA, whereas in up to 38% it can occur in open techniques [6]. A possible explanation for this might be the extra-articular osteotomy site in MICA and sometimes a slightly pronounced shortening of the first metatarsal due to the diameter of the burr. Nevertheless, the shortening can easily be avoided by placing the osteotomy plane to distal–lateral and not perpendicular to the first metatarsal shaft. In the present study, we found significant stiffness in only three cases of the whole cohort at final follow-up; hence, it can be concluded that this complication is extremely rare. This is in accordance with findings in the literature [7, 12].

Our study has some limitations. First, it is a retrospective case series with a relatively small sample size, although the number of patients is well comparable to other studies dealing with MIS hallux valgus correction [10]. Second, the follow-up with 12 months is short. Nevertheless, it can be stated that after 12 months, a consolidation of the clinical result has occurred and roughly 95% of all patients were satisfied with the surgery. Third, we did not use a clinical established patient reported outcome measure (PROM) to evaluate clinical satisfaction, which makes comparison of our clinical results to these of other authors difficult. Although PROMs may be valuable in comparison of various surgical treatments and differences between distinct population groups, clinical interpretation of these differences can sometimes be misleading [14]. Meaning, a statistically significant change with a *p* value < 0.05 in scores may not necessarily translate into a considerable change for patients clinically [17]. To get a clear idea of whether patients were happy or not with the surgery and would opt for it again or against we chose to ask the above mentioned simple questions, and found it very helpful in the clinical setting. Nonetheless, these limitations must be considered before conclusions are drawn about daily practical actions.

## Conclusions

This study proves that third-generation MICA is a very safe procedure, which offers a stable osteosynthesis and, due to the extra-articular osteotomy level, a very good postoperative mobility of the MTPJ. Placement of a second metatarsal screw may not be necessary if intraoperative stability of the metatarsal osteotomy is present. Nevertheless, if any doubt regarding stability of the metatarsal osteotomy is encountered, a second screw should always be inserted, in accordance to orthopedic philosophy: “in dubio, pro bolzo”.

## Data Availability

Primary data can be asked by the corresponding author.

## References

[1] Aiyer A, Massel DH, Siddiqui N et al (2021) Biomechanical comparison of 2 common techniques of minimally invasive hallux valgus correction. Foot Ankle Int 42(3):373–380. https://www.ncbi.nlm.nih.gov/pubmed/33161779 10.1177/1071100720959029 33161779

[2] Altenberger S, Kriegelstein S, Gottschalk O et al (2018) The minimally invasive Chevron and Akin osteotomy (MICA). Oper Orthop Traumatol 30(3):148–160. https://www.ncbi.nlm.nih.gov/pubmed/29671022 10.1007/s00064-018-0541-0 29671022

[3] Bernasconi A, Rizzo M, Izzo A et al (2021) Bosch osteotomy for hallux valgus correction: results at a mean 10-year follow-up. Arch Orthop Trauma Surg. https://www.ncbi.nlm.nih.gov/pubmed/34839385 10.1007/s00402-021-04259-3 34839385

[4] Brogan K, Lindisfarne E, Akehurst H et al (2016) Minimally invasive and open distal Chevron osteotomy for mild to moderate hallux valgus. Foot Ankle Int 37(11):1197–1204. https://www.ncbi.nlm.nih.gov/pubmed/27381179 10.1177/1071100716656440 27381179

[5] Cazeau C, Stiglitz Y (2018) Minimally invasive and percutaneous surgery of the forefoot current techniques in 2018. Eur J Orthop Surg Traumatol 28(5):819–837. https://www.ncbi.nlm.nih.gov/pubmed/29574577 10.1007/s00590-018-2137-7 29574577

[6] Frigg A, Zaugg S, Maquieira G et al (2019) Stiffness and range of motion after minimally invasive Chevron-Akin and open scarf-Akin procedures. Foot Ankle Int 40(5):515–525. https://www.ncbi.nlm.nih.gov/pubmed/30688526 10.1177/1071100718818577 30688526

[7] Hernandez-Castillejo LE, Martinez Vizcaino V, Garrido-Miguel M et al (2020) Effectiveness of hallux valgus surgery on patient quality of life: a systematic review and meta-analysis. Acta Orthop 91(4):450–456. https://www.ncbi.nlm.nih.gov/pubmed/32408787 10.1080/17453674.2020.1764193 PMC8023907 32408787

[8] Izzo A, Vallefuoco S, Basso MA et al (2022) Role of lateral soft tissue release in percutaneous hallux valgus surgery: a systematic review and meta-analysis of the literature. Arch Orthop Trauma Surg. https://www.ncbi.nlm.nih.gov/pubmed/36352268 10.1007/s00402-022-04693-x PMC10293432 36352268

[9] Ji L, Wang K, Ding S et al (2022) Minimally invasive vs. open surgery for hallux valgus: a meta-analysis. Front Surg 9:843410. https://www.ncbi.nlm.nih.gov/pubmed/35388365 10.3389/fsurg.2022.843410 PMC8978717 35388365

[10] Malagelada F, Sahirad C, Dalmau-Pastor M et al (2019) Minimally invasive surgery for hallux valgus: a systematic review of current surgical techniques. Int Orthop 43(3):625–637. https://www.ncbi.nlm.nih.gov/pubmed/30218181 10.1007/s00264-018-4138-x 30218181

[11] Munro BH (2005). Statistical methods for health care research.

[12] Redfern D, Vernois J, Legre BP (2015) Percutaneous surgery of the forefoot. Clin Podiatr Med Surg 32(3):291–332. https://www.ncbi.nlm.nih.gov/pubmed/26117570 10.1016/j.cpm.2015.03.007 26117570

[13] Singh MS, Khurana A, Kapoor D et al (2020) Minimally invasive vs open distal metatarsal osteotomy for hallux valgus—a systematic review and meta-analysis. J Clin Orthop Trauma 11(3):348–356. https://www.ncbi.nlm.nih.gov/pubmed/32405192 10.1016/j.jcot.2020.04.016 PMC7211908 32405192

[14] Singh V, Fiedler B, Huang S et al (2022) Patient acceptable symptom state for the forgotten joint score in primary total knee arthroplasty. J Arthroplasty 37(8):1557–1561. https://www.ncbi.nlm.nih.gov/pubmed/35346809 10.1016/j.arth.2022.03.069 35346809

[15] Toepfer A, Strassle M (2022) 3rd generation MICA with the "K-wires-first technique"—a step-by-step instruction and preliminary results. BMC Musculoskelet Disord 23(1):66. https://www.ncbi.nlm.nih.gov/pubmed/35042485 10.1186/s12891-021-04972-5 PMC8767719 35042485

[16] Toepfer A, Strassle M (2022) The percutaneous learning curve of 3rd generation minimally-invasive Chevron and Akin osteotomy (MICA). Foot Ankle Surg. https://www.ncbi.nlm.nih.gov/pubmed/35882575 10.1016/j.fas.2022.07.006 35882575

[17] Tubach F, Ravaud P, Baron G et al (2005) Evaluation of clinically relevant states in patient reported outcomes in knee and hip osteoarthritis: the patient acceptable symptom state. Ann Rheum Dis 64(1):34–37. https://www.ncbi.nlm.nih.gov/pubmed/15130902 10.1136/ard.2004.023028 PMC1755171 15130902

[18] Vernois J, Redfern DJ (2016) Percutaneous surgery for severe hallux valgus. Foot Ankle Clin 21(3):479–493. https://www.ncbi.nlm.nih.gov/pubmed/27524702 10.1016/j.fcl.2016.04.002 27524702

